# An Integrated Navigation Method Based on the Strapdown Inertial Navigation System/Scene-Matching Navigation System for UAVs

**DOI:** 10.3390/s25113379

**Published:** 2025-05-27

**Authors:** Yukun Wang, Qiang Wang, Zhonghu Hao, Puhua Chen

**Affiliations:** 1 School of Mechatronical Engineering, Beijing Institute of Technology, Beijing 100081, China; 3120185169@bit.edu.cn (Y.W.); haozhonghu@bit.edu.cn (Z.H.); 2 The Key Laboratory of Intelligent Perception and Image Understanding of Ministry of Education, School of Artificial Intelligence, Xidian University, Xi’an 710071, China; phchen@xidian.edu.cn

**Keywords:** SINS, SMNS, KF, integrated navigation system, UAV

## Abstract

To address the challenges of discontinuous heterogeneous image matching, significant matching errors in specific regions, and poor real-time performance in GNSS-denied environments for unmanned aerial vehicles (UAVs), we propose an integrated navigation method based on the strapdown inertial navigation system (SINS)/scene-matching navigation system (SMNS). First, we designed a heterogeneous image-matching and positioning approach using infrared images to obtain an estimation of the UAV’s position. Then, we established a mathematical model for the integrated SINS/SMNS navigation system. Finally, a Kalman filter (KF) was employed to fuse the inertial navigation data with absolute position data from scene matching, achieving high-precision and highly reliable navigation positioning. We constructed a navigation data acquisition platform and conducted simulation studies using flight data collected from this platform. The results demonstrate that the integrated SINS/SMNS navigation method significantly outperforms standalone scene-matching navigation in horizontal positioning accuracy, improving latitude accuracy by 52.34% and longitude accuracy by 45.54%.

## 1. Introduction

Unmanned aerial vehicles (UAVs) have rapidly emerged as a significant technology, attracting considerable attention from various countries. They have been extensively applied across diverse fields, including agriculture, military, and meteorology, significantly influencing the development of these sectors [1]. In the military domain, UAV applications primarily encompass aerial reconnaissance, terminal guidance for cruise navigation, and more [2]. With the rise of the low-altitude economy, the demand for UAVs in the civilian sector is increasing sharply. Beyond surveillance, management, emergency rescue, and climate regulation, emerging application scenarios for UAVs are being continuously developed. In UAV systems, the navigation and positioning subsystem is one of the most critical components, as its performance directly impacts flight safety and mission success.

At present, the most well-developed and effective UAV navigation and positioning system relies on the integration of satellite navigation and inertial navigation technologies [3]. However, satellite navigation can become unreliable in challenging terrains such as mountains or valleys. Moreover, frequent interference with satellite signals during wartime or conflicts significantly degrades the performance of satellite navigation [4]. Inertial navigation systems, on the other hand, suffer from error accumulation over time, which limits their ability to support independent long-term navigation for UAVs. F. D’Ippolito et al. [5] propose a hybrid observer that fuses inertial data with sporadic position updates, achieving fast and robust estimation with formal ISS guarantees. In [6], a vision-based localization method is introduced, where UAV geolocalization is achieved through image matching between onboard camera frames and orthophotos, without requiring GNSS or external infrastructure. Previous research [7] categorizes visual positioning methodologies for UAV navigation in GNSS-deprived scenarios into two principal classifications—Relative Vision Localization (RVL) and Absolute Vision Localization (AVL)—which adopt varied approaches to exploit imaging data for positional estimation. Recent analyses highlight sensor fusion as critical for UAV navigation in GNSS-denied settings. Tong et al. [8] systematically evaluate SLAM architectures and swarm visual localization, demonstrating how the synergistic fusion of visual–inertial, LiDAR–inertial, and LiDAR–visual configurations overcomes single-modality limitations. The SMNS, characterized by its simple structure, strong environmental adaptability, and robust implementation capability, has proven to be an excellent navigation solution in GNSS-denied environments [9].

To successfully implement an SMNS, the following sequential steps need to be undertaken: (1) acquiring and preprocessing infrared images and reference satellite maps to enable image registration; (2) selecting the matching region of interest (ROI) areas based on the pre-planned trajectory of the UAV or the real-time inertial position; (3) performing image matching, through which the geographic coordinate information of key points within the matching ROI areas is obtained; (4) estimating the UAV attitude, including visual position calculation, attitude determination, and correcting inertial navigation deviations through inertial data fusion [10]. Among these steps, image matching is the critical step that directly influences the accuracy of UAV attitude estimation.

Multi-modal image matching is a crucial technology that addresses image matching challenges arising from various image acquisition sources in the SMNS [11]. Generally, there are two paradigms for multi-modal image registration: the first paradigm is the feature-based matching method, which involves feature extraction, feature description, and feature matching; the second paradigm is image matching via template matching [12]. Based on the types of features extracted, feature matching techniques can be categorized into methods based on point features and methods based on structural features. Among these, point feature-based matching methods require less computational effort, making them easier and more efficient to implement. As a result, they are particularly suitable for hardware environments with limited resources. Consequently, numerous studies have focused on such methods under conditions of constrained hardware computing performance in the past, including SIFT (Scale-Invariant Feature Transform), SURF (Speeded-Up Robust Features) [13], FAST (Features from Accelerated Segment Test) [14], BRIEF (Binary Robust Independent Elementary Features) [15], and ORB (Oriented FAST and Rotated BRIEF) [16].

The classical features mentioned above are primarily extracted at the pixel gray level, making them highly susceptible to illumination variations. However, they are well suited for image matching in scenarios with minimal gray-level changes between successive frames. Due to their robustness, these classical features are widely utilized in visual odometry, SLAM, UAV optical flow, and other related applications. In recent years, many researchers have focused on improving these methods based on classical features to achieve multi-modal image matching. To mitigate the impact of nonlinear radiation distortion (NRD), the nonlinear diffusion scale space was introduced in PSO-SIFT [17]. This approach employs a nonlinear diffusion filter and multi-scale parameters to extract uniform gradient information between Synthetic Aperture Radar (SAR) and visible images. Deng et al. [18] proposed a two-step matching approach combining global and local matching to obtain more control points for SIFT-like very-high-resolution SAR image registration, significantly improving the number and quality of matched control points. Zhang et al. [19] combined SIFT with Canny edge detection to remove unstable points and smooth SAR images, significantly enhancing registration accuracy and speed using FLANN and PROSAC. Wu et al. [20] proposed an improved ORB algorithm using affine transformation to extract stable descriptor bits, improving matching accuracy and employing an enhanced F-SORT for refined matching. Zhang et al. [21] proposed an OS-SIFT-based method with a cascaded sample consensus approach for robust optical and SAR image registration, improving gradient consistency and increasing correct correspondences to enhance registration accuracy and robustness. Nehme et al. [22] proposed a deep learning-based wavefront-shaping method that optimizes the optical parameters of a multi-channel imaging system, thereby enhancing image resolution and feature extraction capabilities. Jhan et al. [23] introduced a normalized SURF (N-SURF) method for multi-spectral image matching, significantly increasing the number of correct matching points. The effectiveness of N-SURF was remarkable, with the number of correct matches being several times or even an order of magnitude higher than that of the traditional SURF method. In addition to the classical feature-based methods mentioned above, methods based on structural feature extraction exhibit stronger robustness in image matching. Li et al. [24] proposed a Radiation-Variation-Insensitive Feature Transform (RIFT) which utilizes phase congruency for matching and demonstrates greater robustness compared to traditional methods. Yu et al. [25] introduced a novel consistent feature transform (NCFT), designed to address the significant NRD problem between multi-modal images, aiming to extract rich and robust features.

Yao et al. [26] proposed a space-matching method based on a co-occurrence filter, which can effectively weaken or eliminate NRD while extracting richer structural features in the co-occurrence space. Rouse et al. [27] introduced a structural similarity (SSIM) metric for image quality assessment. SSIM compares the luminance, contrast, and structural information of images, enabling a more accurate reflection of human visual system perception of image quality. Additionally, Yao et al. [28] proposed the Multi-Orientation Tensor Index Feature (MOTIF) for the registration of Synthetic Aperture Radar (SAR) images and optical images. By extracting structural information from multiple orientations, MOTIF significantly reduces the impact of inherent speckle noise in SAR images on the registration process. This method demonstrates strong robustness in the task of SAR and optical image registration, effectively improving registration accuracy. Furthermore, research based on the histogram of absolute phase consistency gradients (HAPCG) [29,30] introduced an anisotropic weighted moment diagram. This diagram effectively extracted edge extreme points from the images and enhanced the number of matching pairs by utilizing the absolute phase direction to determine the main direction and generate characteristic descriptors. These algorithms have significantly advanced research on multi-modal remote sensing image matching. However, their applicability is limited by various factors, including geographical location, scale, rotation, and computational complexity.

The second method to solve the matching problem is template matching based on grayscale correlation or structural similarity. Early image registration methods primarily focused on pixel-gray-level similarity [31]. These methods assess image similarity based on pixel gray levels, making them unsuitable for multi-modal image registration. Currently, robust template matching techniques primarily utilize the structural features of images.

These methods better reflect the common properties of multi-modal images, such as gradients and tensors [32]. Therefore, Ye et al. [33] proposed a matching method based on structural features by introducing the concept of the Histogram of Oriented Phase Congruency (HOPC). An improved HOPC algorithm, called Channel Features of Oriented Gradients (CFOG) [34], was proposed to enhance efficiency by characterizing image structure features pixel by pixel, and template matching was accelerated using the Fourier transform. Ruslan et al. [35] introduced a structure tensor-based multi-modal image-matching method for unmanned aerial vehicle (UAV) scene-matching systems. By extracting the structural tensor features of images, this method can effectively handle illumination variations and demonstrates high robustness and accuracy in multi-modal image matching.

Parallax is calculated based on the positional differences in matching points across different images. Since parallax is related to the distance between the object and the camera, as well as the camera’s attitude, it can be used to derive information about scene depth and changes in the drone’s relative position. By integrating the strapdown inertial navigation system (SINS) [36] with the scene-matching navigation system (SMNS), the UAV can ensure the completion of regular flight tasks even in the absence of satellite positioning signals.

**Figure 1:**
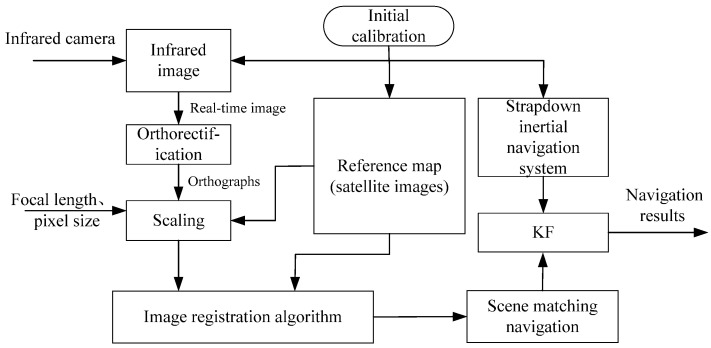
Schematic diagram of SINS/SMNS integrated navigation.

To address the positioning challenges of UAVs in GNSS-denied environments, we propose an integrated navigation framework based on SINS/SMNS fusion. A schematic diagram of the proposed method is illustrated in Figure 1. The key methodological advancements of this work are threefold:

1. A real-time infrared image orthorectification technique based on SINS data is introduced to reduce the impact of UAV attitude on image matching. This method features simple operation and high computational efficiency, delivering excellent real-time performance while achieving optimal processing results.
2. A Log-Gabor [37] filter-based feature extraction method is proposed to extract the structural features of images, addressing the critical challenges of multi-modal image matching and achieving highly robust matching results between real-time images and reference images.
3. A cascaded Kalman filtering [38] mechanism is designed to integrate high-frequency SINS measurements with SMNS positional updates. This fusion strategy reduces cumulative errors by 52.34% in latitude and 45.54% in longitude compared to standalone SMNS implementations.

Through the above procedures, the UAV can achieve long-term stable navigation performance. To validate the real-world performance of the proposed method, extensive simulation experiments were conducted using real flight data. The experimental results further confirm the effectiveness and practicality of the proposed method.

The remainder of this paper is organized as follows: Section 2, Section 3 and Section 4 present the three main procedures of the proposed method, while Section 5 provides the experimental results and analysis. Finally, Section 6 concludes the paper with a summary of the work.

## 2. Orthorectification of Oblique Images Based on Inertial Attitude

In general, scene-matching navigation systems utilize satellite images derived from orthophotos as the reference map. However, real-time images captured by the UAV at different attitude angles during flight exhibit significant geometric distortion. This distortion substantially impacts the matching accuracy between the real-time image and the reference satellite image. Therefore, it is essential to correct the real-time image into an orthophoto using the camera installation error, UAV attitude, and camera-intrinsic parameters prior to real-time scene matching.

The coordinate frames used in this paper are defined as follows:

1. ***OX_b_Y_b_Z_b_***: UAV body frame. The x-axis points to the right wing, the y-axis points to the front of the UAV, and the z-axis points vertically upward.
2. ***O*′*X_c_Y_c_Z_c_***: camera frame. ***O*′** represents the optical center of the camera, which may not coincide with the body-frame center ***O***. The x-axis points to the right wing, the y-axis points to the tail of the UAV, and the z-axis points vertically downward.
3. ***OX_g_Y_g_Z_g_***: local gravity frame. The origin is centered at the UAV’s center of mass. The x-axis points geographically east, the y-axis points geographically north, and the z-axis points vertically upward, perpendicular to the local reference ellipsoid surface, and is almost opposite to the direction of gravity.

The camera is rigidly mounted on the UAV, with ***O*′*Z_c_*** aligned parallel to the normal axis of the UAV and oriented perpendicular to the UAV body, pointing downward. This configuration ensures that the real-time ground scene can be continuously captured during the UAV’s movement.

**Figure 2:**
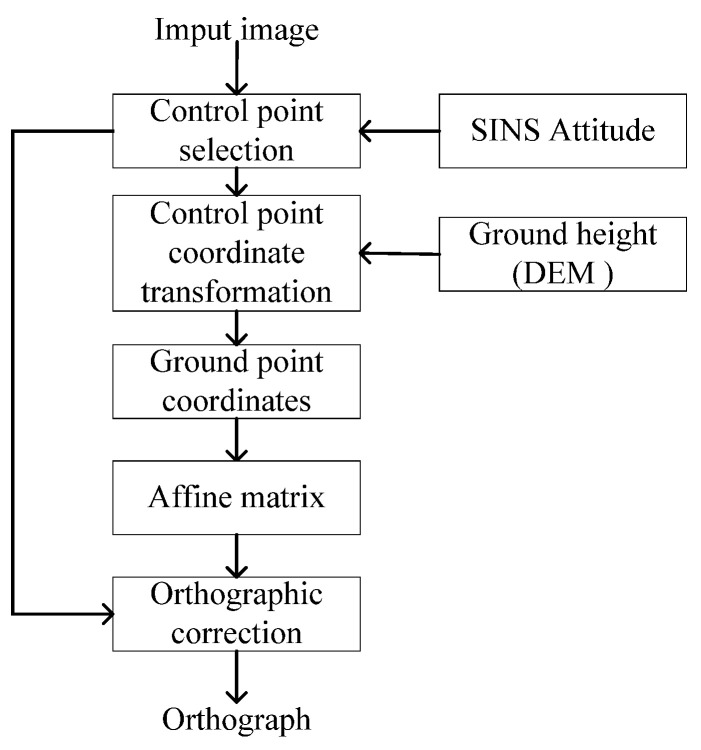
Automatic orthorectification process with SINS and digital elevation model (DEM).

In this study, we utilize attitude information from a high-precision laser inertial navigation system to assist in the orthorectification of images, with the specific operations illustrated in Figure 2. Firstly, we identify the control points within the UAV-captured real-time images, and their positions in the camera frame are computed based on the intrinsic parameters of the camera. Given that the camera is co-aligned with the UAV, the attitude matrix enables the projection of these control points into the local gravity frame. Subsequently, within the local gravity frame, the positions of the control points relative to the UAV can be determined by using the triangle similarity principle, enabling the calculation of their geographical positions.

**Figure 3:**
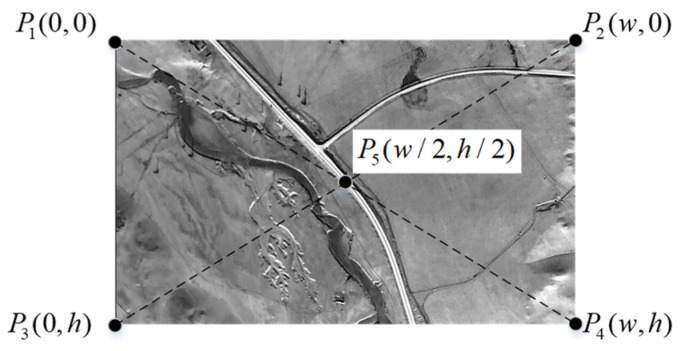
The coordinates of the control points (specifically the four corner points and the center point).

The coordinates of the control points are illustrated in Figure 3. The image resolution is w×h, where w represents the pixel width of the image, and h represents the pixel height of the image. The four corner points and the center point are selected as the control points, denoted as $P_{i}(x_{i},y_{i})$ (where *i* = 1, 2, …, 5). Their coordinates in the pixel frame are as follows: $P_{1}(0,\;0)$, $P_{2}\left(w,\;0\right)$, $P_{3}(0,\;h)$, $P_{4}(w,\;h)$, and $P_{5}(w/2,\;h/2)$.

The coordinates of the control points in the camera frame can be expressed as follows:

(1) $$P_{ci}=M_{pc}*\left[\begin{array}{c} P_{i}\\ 1 \end{array}\right]=M_{pc}*\left[\begin{array}{c} x_{i}\\ y_{i}\\ 1 \end{array}\right]$$

(2) $$M_{pc}=\left[\begin{array}{ccc} P_{xy} & 0 & -\frac{w}{2}*P_{xy}\\ 0 & P_{xy} & -\frac{h}{2}*P_{xy}\\ 0 & 0 & f \end{array}\right]$$

In these equations, $P_{ci}$ represents the coordinates of the control points in the camera frame, $M_{pc}$ is the transformation matrix mapping the control point positions to the camera frame, $P_{xy}$ denotes the camera sensor’s pixel size (unit: mm/pixel), and *f* represents the focal length of the camera.

Since the distortion of industrial cameras is typically very small and usually unknown, the impact of camera distortion on orthographic images can be essentially neglected. Therefore, the positions of the control points in the camera frame are determined using the intrinsic parameters of the camera.

Denoting the pitch angle of the UAV by *θ*, the roll angle by ϕ, and the yaw angle by ψ, the direction cosine matrix R of the UAV can be expressed as Equation (3):

(3) $$R=\left[\begin{array}{ccc} \cos\phi \cos\psi +\sin\phi \sin\theta \sin\psi & \cos\theta \sin\psi & \sin\phi \cos\psi -\cos\phi \sin\theta \sin\psi \\ -\cos\phi \sin\psi +\sin\phi \sin\theta \cos\psi & \cos\theta \cos\psi & -\sin\phi \sin\psi -\cos\phi \sin\theta \cos\psi \\ -\sin\phi \cos\theta & \sin\theta & \cos\phi \cos\theta \end{array}\right]$$

The camera and UAV are rigidly mounted, and the control points $P_{gi}$ in the local gravity frame can be calculated using the matrix R. It can be expressed as follows:

(4) $$P_{gi}=\left[\begin{array}{c} x_{gi}\\ y_{gi}\\ z_{gi} \end{array}\right]=R^{-1}P_{ci}=R^{-1}\left[\begin{array}{c} x_{ci}\\ y_{ci}\\ f \end{array}\right]$$

According to the definition of the local gravity frame, the z-axis is perpendicular to the ground and opposite to the direction of gravity. The intersection point of the z-axis with the ground represents the relative flight height of the UAV, denoted as $h_{r}$. Assuming that the flight altitude of the UAV is $h_{b}$, the elevation of the ground point directly below the UAV can be obtained from the DEM, denoted as $h_{gi}$. The relative height of the control point is denoted as $z_{di}$, and $z_{di}=h_{r}=h_{b}-h_{gi}$. Therefore, the position of the control point in the local gravity frame can be calculated based on the triangle similarity principle, i.e.,

(5) $$P_{di}=\frac{z_{di}}{z_{gi}}P_{gi}=\frac{h_{b}-h_{gi}}{z_{gi}}P_{gi}=\left[\begin{array}{c} \frac{x_{gi}}{z_{gi}}(h_{b}-h_{gi})\\ \frac{y_{gi}}{z_{gi}}(h_{b}-h_{gi})\\ h_{b}-h_{gi} \end{array}\right]$$

Since *R* is a unit orthogonal matrix, we can assume that

(6) $$R^{-1}=\left[\begin{array}{ccc} r_{11} & r_{12} & r_{13}\\ r_{21} & r_{22} & r_{23}\\ r_{31} & r_{32} & r_{33} \end{array}\right]$$

Based on Equations (1), (2) and (4), it can be inferred that

(7) $$z_{gi}=\left(x_{i}-w/2\right)P_{xy}r_{31}+\left(y_{i}-h/2\right)P_{xy}r_{32}+r_{33}f$$

Based on Equations (1)–(6), Equation (8) can be inferred as follows:

(8) $$\begin{array}{cc} P_{di} & =\frac{z_{di}}{z_{gi}}R^{-1}M_{pc}\left[\begin{array}{c} P_{i}\\ 1 \end{array}\right]\\ & =\frac{h_{b}-h_{gi}}{z_{gi}}\left[\begin{array}{ccc} r_{11} & r_{12} & r_{13}\\ r_{21} & r_{22} & r_{23}\\ r_{31} & r_{32} & r_{33} \end{array}\right]\left[\begin{array}{ccc} P_{xy} & 0 & -(w/2)P_{xy}\\ 0 & P_{xy} & -(h/2)P_{xy}\\ 0 & 0 & f \end{array}\right]\left[\begin{array}{c} x_{i}\\ y_{i}\\ 1 \end{array}\right]\\ & =(h_{b}-h_{gi})\left[\begin{array}{c} \frac{(x_{i}-w/2)P_{xy}r_{11}+(y_{i}-h/2)P_{xy}r_{12}+r_{13}f}{(x_{i}-w/2)P_{xy}r_{31}+(y_{i}-h/2)P_{xy}r_{32}+r_{33}f}\\ \frac{(x_{i}-w/2)P_{xy}r_{21}+(y_{i}-h/2)P_{xy}r_{22}+r_{23}f}{(x_{i}-w/2)P_{xy}r_{31}+(y_{i}-h/2)P_{xy}r_{32}+r_{33}f}\\ 1 \end{array}\right] \end{array}$$

To facilitate image registration, it is essential to ensure that the scale of the orthophoto should match that of the reference satellite image. And the resolution of the orthograph should be the same as the reference satellite image. The resolution accuracy of the reference map can be expressed as $m_{res}$, and the scaling and translation operations on the control points can be computed as shown in Equation (9).

(9) $$P_{ni}=\frac{P_{di}-P_{d5}}{m_{res}},i=1,\;2,\;3,\;4$$

The affine matrix can be calculated using the Gaussian elimination method by designating the four corners of the image as control points. Assuming that the matrix is denoted as $M_{A}$, it can be formulated as follows:

(10) $$M_{A}=\left[\begin{array}{ccc} a & b & c\\ d & e & f\\ 0 & 0 & 1 \end{array}\right]$$

(11) $$P_{ni}=M_{A}\left[\begin{array}{c} P_{i}\\ 1 \end{array}\right]=M_{A}\left[\begin{array}{c} x_{i}\\ y_{i}\\ 1 \end{array}\right]$$

Through the substitution of $P_{ni}$ (where *i* = 1, 2, 3, 4) and $P_{i}$ (where *i* = 1, 2, 3, 4) into the above equations, the solution can be obtained using the Gaussian elimination method. Consequently, the orthographic image can be generated by applying the affine matrix $M_{A}$.

## 3. Image Registration Method Based on Maximum Index Map

In image matching, structural features are more suitable for multi-modal image matching. The template-matching method exhibits greater robustness compared to feature point-based methods, particularly when the scale of the template image aligns with that of the reference image. Additionally, the template-matching approach eliminates the need for complex steps, such as RANSAC, significantly enhancing the overall robustness of the system. Therefore, in this work, we adopt the template-matching [39] approach to achieve image matching. The image-matching process comprises two main steps:

1. Calculate the structural feature maps of the infrared image and the satellite map using the Log-Gabor filter;
2. Utilize template matching to achieve automatic image matching.

### 3.1. Log-Gabor Filter

Compared with the Gabor filter, the Log-Gabor filter can effectively capture the local frequency-domain information of an image. Moreover, its bandwidth and center frequency can be manually configured. The expression is as follows:

(12) $$G(\omega )=e^{\frac{-log(\omega /\omega_{0}{)}^{2}}{2\log((k/\omega_{0}{)}^{2})}}$$

In this equation, $\omega_{0}$ represents the center frequency of the filter. To obtain constant shape ratio filters, the term $k/\omega_{0}$ must remain constant for varying $\omega_{0}$.

A Two-Dimensional Log-Gabor filter (2D-LGF) consists of two components—a radial filter $G_{r}(r)$ and an angle filter $G_{\theta }(\theta )$—both of which are expressed in Gaussian form:

(13) $$\left\{\begin{array}{c} G_{r}(r)=\exp(-\frac{log(r/f_{0}{)}^{2}}{2\sigma_{r}^{2}})\\ G_{\theta }(\theta )=\exp(-\frac{(\theta -\theta_{0}{)}^{2}}{2\sigma_{\theta }^{2}}) \end{array}\right.$$

(14) $$G(r,\theta )=G_{r}(r)G_{\theta }(\theta )$$

In this equation, r and θ represent the radial and angular components, respectively; $\sigma_{r}$ denotes the radial bandwidth and $\sigma_{\theta }$ represents the angular bandwidth of the filter; and $f_{0}$ and $\theta_{0}$ denote the center frequency and angular direction, respectively. The expression for $f_{0}$ is as follows:

(15) $$f_{0}=\frac{1}{\omega^{k}*m_{t}^{s-1}}$$

In this equation, ω represents the frequency parameter, k is a constant typically associated with the filter design, $m_{t}$ denotes the scale parameter, and s is the scale index.

The Log-Gabor filter can only be expressed in the frequency domain, while its spatial-domain representation is expressed in complex form. Through the inverse Fourier transform, the 2D-LGF can be expressed as follows:

(16) $$G_{so}(x,y)=G_{so}^{e}(x,y)+iG_{so}^{o}(x,y)$$

In this equation, $G_{so}^{e}$ and $G_{so}^{o}$ represent the even-symmetric and odd-symmetric filters in terms of scale and direction, respectively. Additionally, $G_{so}^{e}$ and $G_{so}^{o}$ are a pair of orthogonal filters.

### 3.2. Maximum Index Map (MIM)

**Figure 4:**
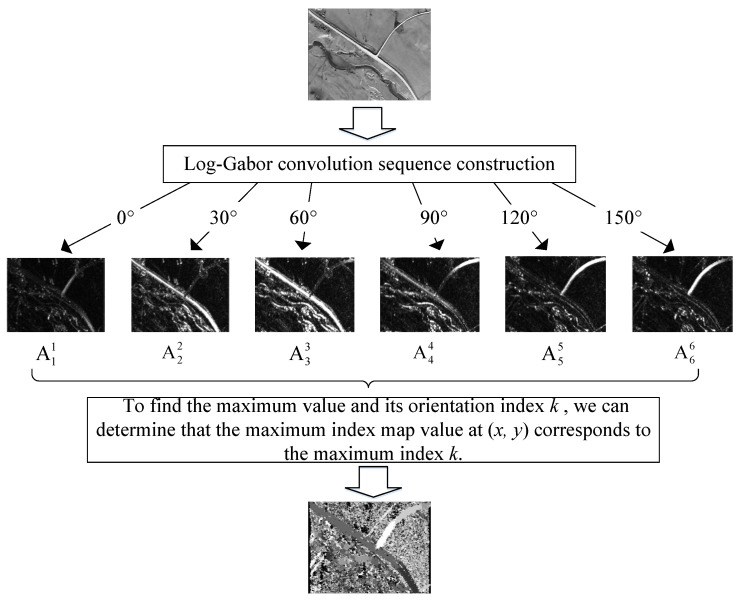
Multi-scale Log-Gabor filtering pipeline for maximum index map generation.

The MIM [40] is constructed using the Log-Gabor filter. For an image M, M(x,y) represents the pixel value at coordinates (x,y) (Figure 4). The convolution components of the even part $E_{so}(x,y)$ and the odd part $O_{so}(x,y)$ can be obtained by applying a 2D-LGF:

(17) $$\left[E_{so}(x,y),O_{so}(x,y)\right]=\left[M(x,y)*G_{so}^{E},M(x,y)*G_{so}^{O}\right]$$

For a given orientation o and scale s, $G_{so}^{O}$ denotes the odd-symmetric filter, and $G_{so}^{E}$ denotes the even-symmetric filter. The phase $\phi_{so}(x,y)$ and amplitude $A_{so}(x,y)$ of M(x,y) are computed as follows:

(18) $$\phi_{so}(x,y)=\mathrm{atan}2(E_{so}(x,y),O_{so}(x,y))$$

(19) $$A_{so}(x,y)=\sqrt{E_{so}(x,y{)}^{2}+O_{so}(x,y{)}^{2}}$$

For a given orientation o, the amplitudes across all scales are summed to obtain a Log-Gabor layer, expressed as follows:

(20) $$A_{o}(x,y)=\sum A_{so}(x,y)$$

Next, to determine the maximum value $A_{k}(x,y)$ and the corresponding orientation index k, we extract the maximum index map value $I_{MIM}(x,y)$ for the maximum index k, i.e.,

(21) $$I_{MIM}(x,y)=k,\;\mathrm{where}\;A_{k}(x,y)=\max\{A_{o}(x,y)\}$$

The maximum index maps for both the infrared image and the satellite map can be independently computed, effectively mitigating the impact of multi-modal imaging variations caused by differences in illumination and radiation. Subsequently, registration between the infrared image and the satellite map can be achieved using template-matching methods.

### 3.3. Template Matching

Common template-matching methods include the Sum of Squared Differences (SSD) [41,42], the Normalized Correlation Coefficient (NCC) [41,43], and Mutual Information (MI) [44]. The SSD is one of the simplest similarity metrics, as it identifies control points by directly computing intensity differences between two images. However, the SSD is highly sensitive to radiometric changes, despite its computational efficiency. The NCC is a widely used similarity metric, particularly in remote sensing image registration, due to its invariance to linear intensity variations.

**Figure 5:**
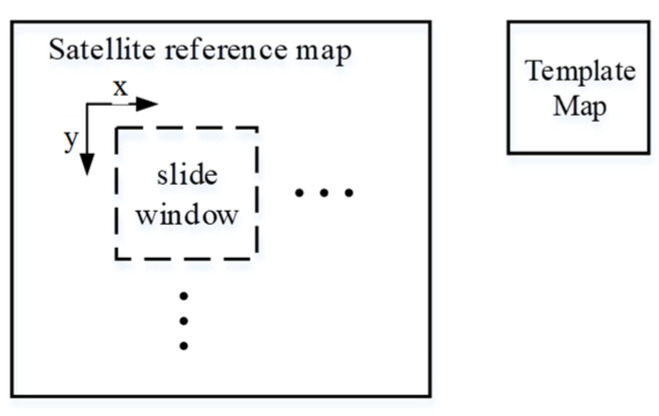
Template-matching process using sliding window search on satellite reference coordinates.

In the template-matching process (Figure 5), the MIM of the infrared image serves as the template, while the MIM of the satellite reference map acts as the reference map. The template is systematically moved from left to right and top to bottom across the reference map. Following the correlation operation, a correlation image is generated, where each pixel value represents the similarity between the infrared image and the satellite reference map.

(22) $$R(x,y)=\frac{\sum_{x’,y’}(T(x’,y’)\cdot I(x+x’,y+y’))}{\sqrt{\sum_{x’,y’}T(x’,y’{)}^{2}\cdot \sum_{x’,y’}I(x+x’,y+y’{)}^{2}}}$$

In this equation, T represents the template and I denotes the reference map, and (x, y) and $\left(x’,y’\right)$ are the horizontal and vertical coordinates of the pixels in the template and reference image, respectively.

According to Equation (22), the position where R(x, y) reaches its maximum value corresponds to the matching point between the infrared image and the reference image. Since the position of each pixel in the reference map is known, the absolute horizontal positions of the four corner points of the real-time image within the reference map can be determined. Additionally, altitude information can be obtained from the drone’s barometer.

In template matching, the confidence level of registration results is inversely proportional to the spread of correlation peaks. This study establishes reliability criteria based on peak dominance analysis: when the secondary maximum correlation coefficient R(x,y) beyond a 15-pixel radius from the primary peak exhibits > 30% attenuation relative to the principal response, the matching result is deemed credible. This threshold-based peak discrimination enables a computationally efficient confidence assessment.

The spatial extent of satellite reference imagery is dynamically adjusted based on temporal proximity to the last successful georegistration event. Frequent successful matches within brief intervals signify sustained positional consistency between the estimated and actual coordinates, enabling constrained search areas. Conversely, prolonged intervals since the last validated match necessitate the progressive expansion of the reference coverage to accommodate potential inertial navigation drift, thereby maintaining reliable alignment between real-time aerial imagery and geospatial reference databases.

So far, we have established four sets of 3D-2D corresponding points, which constitute a Perspective-n-Point (PnP) problem [45]. Based on the PnP problem-solving methodology, the four sets of correspondences can be utilized to derive a unique solution, namely, the UAV’s pose. Consequently, by solving the PnP problem, we ultimately determine the position of the UAV based on the scene-matching approach.

## 4. Integrated Navigation Method Based on SINS/SMNS

Due to the low frequency of image registration, the speed of scene-matching position calculation is significantly reduced. Poor ground texture clarity further exacerbates this issue, leading to suboptimal scene-matching results and substantial errors in position estimation. To address these challenges and improve the positioning accuracy of UAVs in satellite-denied environments, we propose a sensor data fusion method that utilizes a KF to establish a mathematical model for an integrated INS/SMNS navigation system.

Generally, the KF consists of two main steps: state prediction and measurement update. The state prediction is based on the dynamic model of the Inertial Measurement Unit (IMU) and is propagated at a frequency of 200 Hz. When the navigation computer completes the motion estimation, the measurement update is triggered and executed. However, scene matching and pose estimation have been proven to be highly time-consuming processes. Therefore, compensating for the time-delay error in the SINS/SMNS system is essential to ensure the system’s real-time performance.

### 4.1. State Equation Design

The state equation of the integrated navigation system is formulated as follows:

(23) $$\dot{X}(t{)}_{15\times 1}=F(t{)}_{15\times 15}X(t{)}_{15\times 1}+G(t{)}_{15\times 6}W(t{)}_{6\times 1}$$

where $X(t{)}_{15\times 1}$ is the system state vector, $F(t{)}_{15\times 15}$ is the system transition matrix, $G(t{)}_{15\times 6}$ is the system noise driving matrix, and $W(t{)}_{6\times 1}$ is the system zero-mean white noise vector. The system state vector can be expressed as

(24) $$X={\left[\begin{array}{ccccccccccccccc} \phi_{e} & \phi_{n} & \phi_{u} & \delta v_{e} & \delta v_{n} & \delta v_{u} & \delta L & \delta \lambda & \delta h & \varepsilon_{x} & \varepsilon_{y} & \varepsilon_{z} & \nabla_{x} & \nabla_{y} & \nabla_{z} \end{array}\right]}^{T}\;\;\;\;$$

where $\phi_{e}$, $\phi_{n}$, and $\phi_{u}$ represent the misalignment angles; $\delta v_{e}$, $\delta v_{n}$, and $\delta v_{u}$ represent velocity errors; δL, δλ, and δh represent position errors; $\varepsilon_{x}$, $\varepsilon_{y}$, and $\varepsilon_{z}$ represent constant gyro drifts; and $\nabla_{x}$, $\nabla_{y}$, and $\nabla_{z}$ are constant accelerometer offsets.

### 4.2. Measurement Equation Design

The longitude, latitude, and altitude of the SMNS are represented by $\lambda_{sm}$, $L_{sm}$, and $h_{sm}$, respectively. To convert these coordinates from the navigation coordinate system to Earth-Centered, Earth-Fixed (ECEF) coordinates ($X_{sm}$, $Y_{sm}$, $Z_{sm}$), the following transformation formulas can be applied:

(25) $$\left\{\begin{array}{c} X_{sm}=(R_{n}+h_{sm})\cos L_{sm}\cos\lambda_{sm}\\ Y_{sm}=(R_{n}+h_{sm})\cos L_{sm}\sin\lambda_{sm}\\ Z_{sm}=[R_{n}(1-e^{2})+h_{sm}]\sin L_{sm} \end{array}\right.$$

where $R_{n}$ represents the radius of curvature in the prime vertical, and e denotes the eccentricity of the Earth’s ellipsoid.

Let $\lambda_{ins}$, $L_{ins}$, and $h_{ins}$ represent the longitude, latitude, and altitude of the INS, respectively. The transformation from the navigation coordinate system to Earth-Centered, Earth-Fixed (ECEF) coordinates ($X_{ins}$, $Y_{ins}$, $Z_{ins}$) can be performed using the following formulas:

(26) $$\left\{\begin{array}{c} X_{ins}=(R_{n}+h_{ins})\cos L_{ins}\cos\lambda_{ins}\\ Y_{ins}=(R_{n}+h_{ins})\cos L_{ins}\sin\lambda_{ins}\\ Z_{ins}=[R_{n}(1-e^{2})+h_{ins}]\sin L_{ins} \end{array}\right.$$

where $\delta \lambda =\lambda_{ins}-\lambda_{sm}$, $\delta L=L_{ins}-L_{sm}$, and $\delta h=h_{ins}-h_{sm}$ represent the longitude error, latitude error, and altitude error in the navigation coordinate system, respectively. $\delta X=X_{ins}-X_{sm}$, $\delta Y=Y_{ins}-Y_{sm}$, and $\delta Z=Z_{ins}-Z_{sm}$ denote the corresponding errors in the ECEF coordinate system.

Based on Equations (25) and (26), the values of δX, δY, and δZ can be derived as follows:

(27) $$\begin{array}{ll} \delta X & =X_{ins}-X_{sm}=(R_{n}+h_{ins})\cos L_{ins}\cos\lambda_{ins}-(R_{n}+h_{sm})\cos L_{sm}\cos\lambda_{sm}\\ & =(R_{n}+h_{ins})\cos L_{ins}\cos\lambda_{ins}-(R_{n}+h_{ins}-\delta h)\cos\left(L_{ins}-\delta L\right)\cos\left(\lambda_{ins}-\delta \lambda \right)\\ & =(R_{n}+h_{ins})\left[\cos L_{ins}\cos\lambda_{ins}-\cos\left(L_{ins}-\delta L\right)\cos\left(\lambda_{ins}-\delta \lambda \right)\right]+\delta h\cos\left(L_{ins}-\delta L\right)\cos\left(\lambda_{ins}-\delta \lambda \right)\\ & =-(R_{n}+h_{ins})\left[\cos L_{ins}\sin\lambda_{ins}\delta \lambda +\sin L_{ins}\cos\lambda_{ins}\delta L\right]+\delta h\cos L_{ins}\cos\lambda_{ins}+\eta_{X} \end{array}$$

(28) $$\begin{array}{ll} \delta Y & =Y_{ins}-Y_{sm}=(R_{n}+h_{ins})\cos L_{ins}\sin\lambda_{ins}-(R_{n}+h_{sm})\cos L_{sm}\sin\lambda_{sm}\\ & =(R_{n}+h_{ins})\cos L_{ins}\sin\lambda_{ins}-(R_{n}+h_{ins}-\delta h)\cos\left(L_{ins}-\delta L\right)\sin\left(\lambda_{ins}-\delta \lambda \right)\\ & =(R_{n}+h_{ins})\left[\cos L_{ins}\sin\lambda_{ins}-\cos\left(L_{ins}-\delta L\right)\sin\left(\lambda_{ins}-\delta \lambda \right)\right]+\delta h\cos\left(L_{ins}-\delta L\right)\sin\left(\lambda_{ins}-\delta \lambda \right)\\ & =(R_{n}+h_{ins})\left(\cos L_{ins}\cos\lambda_{ins}\delta \lambda -\sin L_{ins}\sin\lambda_{ins}\delta L\right)+\delta h\cos L_{ins}\sin\lambda_{ins}+\eta_{Y} \end{array}$$

(29) $$\begin{array}{ll} \delta Z & =Z_{ins}-Z_{sm}=[R_{n}(1-e^{2})+h_{ins}]\sin L_{ins}-[R_{n}(1-e^{2})+h_{sm}]\sin L_{sm}\\ & =[R_{n}(1-e^{2})+h_{ins}]\sin L_{ins}-[R_{n}(1-e^{2})+h_{ins}-\delta h]\sin\left(L_{ins}-\delta L\right)\\ & =[R_{n}(1-e^{2})+h_{ins}]\left[\sin L_{ins}-\sin\left(L_{ins}-\delta L\right)\right]+\delta h\sin\left(L_{ins}-\delta L\right)\\ & =[R_{n}(1-e^{2})+h_{ins}]\cos L_{ins}\delta L+\sin L_{ins}\delta h+\eta_{Z} \end{array}$$

where $\eta_{X}$, $\eta_{Y}$, and $\eta_{Z}$ represent second-order small error quantities in different directions.

Based on Equations (27)–(29), the measurement equation can be derived as follows:

(30) $$Z_{3\times 1}(t)=\left[\begin{array}{c} X_{ins}-X_{sm}\\ Y_{ins}-Y_{sm}\\ Z_{ins}-Z_{sm} \end{array}\right]=H_{3\times 15}(t)X_{15\times 1}(t)+N_{3\times 1}(t)$$

And $H_{3\times 15}$ can be expressed as follows:

(31) $$H_{3\times 15}=\left[\begin{array}{cccc} 0_{3\times 3} & 0_{3\times 3} & M_{3\times 3} & 0_{3\times 6} \end{array}\right]$$

where $Z_{3\times 1}(t)$ is the measurement vector, $H_{3\times 15}(t)$ is the observation matrix, and ${\;N}_{3\times 1}(t)$ is the measurement noise driving matrix.

Additionally, ${\;M}_{3\times 3}$ can be expressed as follows:

(32) $$M_{3\times 3}=\left[\begin{array}{ccc} -(R_{n}+h_{ins})\sin L_{ins}\cos\lambda_{ins} & -(R_{n}+h_{ins})\cos L_{ins}\sin\lambda_{ins} & \cos L_{ins}\cos\lambda_{ins}\\ -(R_{n}+h_{ins})\sin L_{ins}\sin\lambda_{ins} & (R_{n}+h_{ins})\cos L_{ins}\cos\lambda_{ins} & \cos L_{ins}\sin\lambda_{ins}\\ {}[R_{n}(1-e^{2})+h_{ins}]\cos L_{ins} & 0 & \sin L_{ins} \end{array}\right]$$

### 4.3. Scene-Matching Position Error Compensation

The location for scene matching is obtained through a series of steps, including image acquisition, image processing, image matching, and position calculation. Notably, the time consumed by image processing and image matching often exceeds 500 ms. To ensure the real-time performance of navigation parameters, it is essential to compensate for the time delay in the location results derived from scene matching.

**Figure 6:**
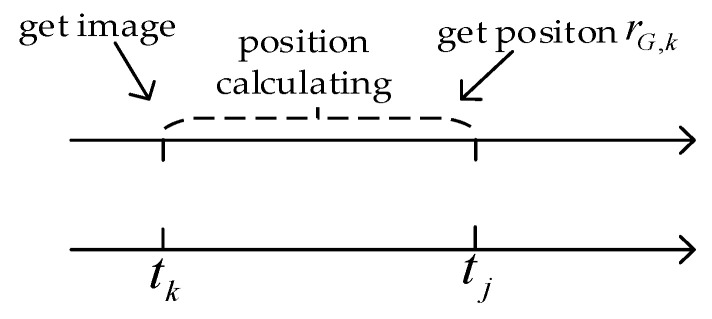
Real-time position compensation framework for scene matching.

As shown in Figure 6, $r_{I,k}$ and $r_{I,j\;}$ are the UAV positions estimated by inertial navigation at times $t_{k}$ and $t_{j}$, respectively. The result of the scene-matching position calculation at time $t_{k}$ is $r_{G,k}$. In reality, the position $r_{G,j}$ at time $t_{j}$ can be approximated as

(33) $$r_{G,j}=r_{G,k}+(r_{I,j}-r_{I,k})$$

### 4.4. Design and Implementation of KF

Through the discretization of the state equation and measurement equation of the integrated navigation system, the discrete system dynamic equation can be derived as follows:

(34) $$\left\{\begin{array}{l} X_{k}=\Phi_{k,k-1}X_{k-1}+\Gamma_{k,k-1}W_{k-1}\\ Z_{k}=H_{k}X_{k}+N_{k} \end{array}\right.$$

In this equation, $\Phi_{k|k-1}$ is the state transition matrix, and τ is the sampling time: $\Phi_{k|k-1}=\sum_{n=0}^{\infty }\frac{{\left[F(t_{k})*\tau \right]}^{n}}{n!}$, $\Gamma_{k,k-1}=\sum_{n=1}^{\infty }\frac{{\left[F(t_{k})*\tau \right]}^{n-1}}{n!}*G(t_{k})*\tau$.

The KF filtering process is described as follows:

(35) $$\left\{\begin{array}{l} {\hat{X}}_{k|k-1}=\Phi_{k|k-1}{\hat{X}}_{k-1}\\ P_{k|k-1}=\Phi_{k|k-1}P_{k-1}\Phi_{k,k-1}^{T}+\Gamma_{k|k-1}Q_{k-1}\Gamma_{k|k-1}^{T}\\ K_{k}=P_{k|k-1}H_{k}^{T}(H_{k}P_{k|k-1}H_{k}^{T}+R_{k}{)}^{-1}\\ {\hat{X}}_{k}={\hat{X}}_{k|k-1}+K_{k}(Z_{k}-H_{k}{\hat{X}}_{k|k-1})\\ P_{k}=(I-K_{k}H_{k})P_{k|k-1}(I-K_{k}H_{k}{)}^{T}+K_{k}R_{k}K_{k}^{T} \end{array}\right.$$

In this equation, $P_{k|k-1}$ is the one-step prediction covariance matrix, $Q_{k-1}$ is the system noise covariance matrix, $K_{k}$ is the filtering gain matrix, and $R_{k}$ is the covariance matrix of the observed value.

## 5. Experimental Results and Analysis

### 5.1. Integrated Navigation System Verification Platform

We have developed an integrated navigation system verification platform. This platform includes a day–night electro-optical reconnaissance payload, a laser inertial navigation system, an atmospheric data sensor system, a Beidou navigation receiver, a data logger, and a high-performance processor. The purpose of this platform is to collect flight data from various sensors to facilitate research on navigation algorithms.

**Figure 7:**
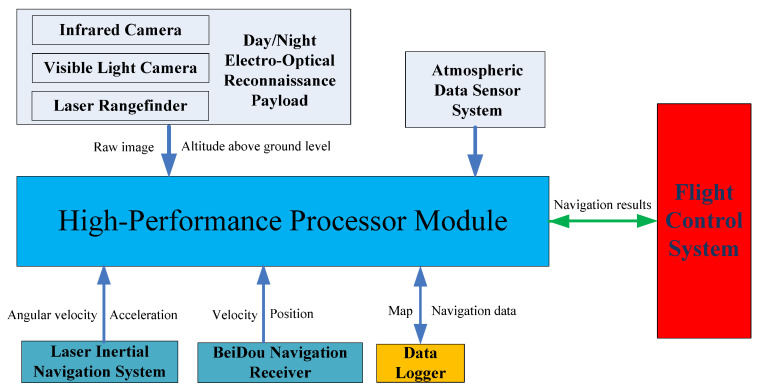
Integrated navigation system verification platform architecture.

**Figure 8:**
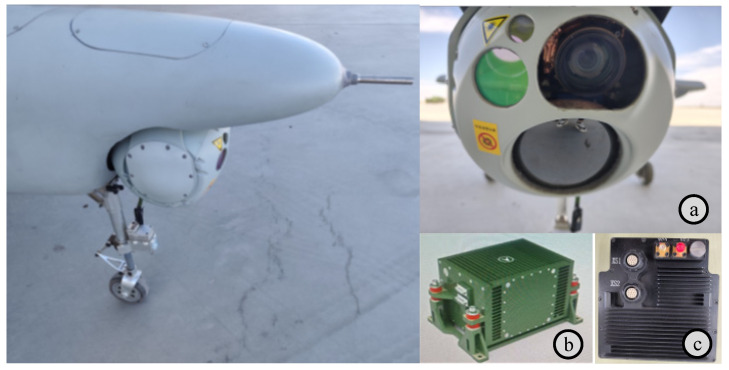
UAV platform and key subsystems: (**a**) Day–night electro-optical reconnaissance payload integrating a visible light camera (1920 × 1080), an infrared thermal imager (1280 × 1024), and a laser rangefinder (5 m accuracy); (**b**) A laser strapdown inertial navigation system (LSINS) with ≤0.05°/h gyro bias; (**c**) A custom high-performance computing module incorporating an NVIDIA Jetson AGX Xavier in a 3D-printed enclosure. The NVIDIA Jetson AGX Xavier is designed and manufactured by NVIDIA (Santa Clara, CA, United States).

The architecture of the UAV integrated navigation verification platform is illustrated in Figure 7. The day–night electro-optical reconnaissance payload is a device integrated with multiple electro-optical sensors, including an infrared camera, a visible light camera, and a laser rangefinder. The visible light camera is used for high-resolution image acquisition during the day, capable of capturing detailed color images. The infrared camera is used for thermal imaging at night or in low-light environments, capable of detecting and recording infrared radiation emitted by objects. The laser rangefinder is used to measure target distances. The laser inertial navigation system provides angular velocity and acceleration information. The Beidou navigation receiver provides velocity and position information. The atmospheric data sensor system provides airspeed and barometric altitude information. The high-performance processor module is used to collect data from various sensors and perform integrated navigation algorithm computations. The flight control system receives data from the integrated navigation system to participate in flight control. Component specifications of the integrated navigation system verification platform are detailed in Table 1 and Figure 8.

### 5.2. Experiments on Orthorectification

**Figure 9:**
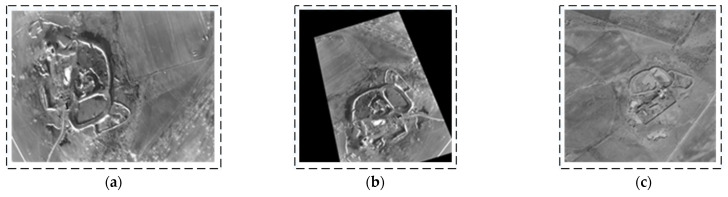
First orthorectification result for UAV in right turn (UAV attitude: yaw = 252.772°; pitch = 1.172°; roll = 24.375°). (**a**) Original image (long-wave infrared image). (**b**) Orthorectified image (scaled to match reference figure); (**c**) satellite reference map (satellite images sourced from ArcGIS and stored onboard UAV).

**Figure 10:**
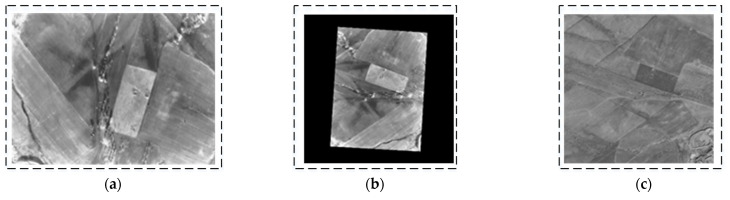
Second orthorectification result for UAV in level flight (UAV attitude: yaw = 273.507°; pitch = 2.093°; roll = 0.549°). (**a**) Original image (long-wave infrared image). (**b**) Orthorectified image (scaled to match reference figure). (**c**) Satellite reference map (satellite images sourced from ArcGIS and stored onboard UAV).

**Figure 11:**
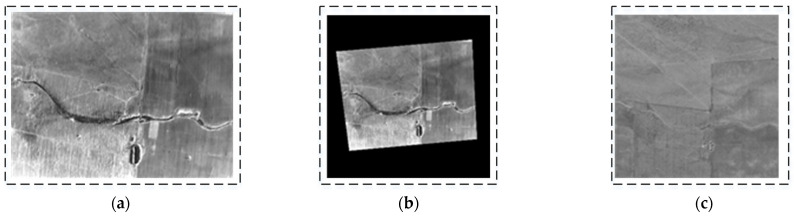
Third orthorectification result for UAV in climbing flight (UAV attitude: yaw = 354.88°; pitch = 9.116°; roll = 0.397°). (**a**) Original image (long-wave infrared image). (**b**) Orthorectified image (scaled to match reference figure). (**c**) Satellite reference map (satellite images sourced from ArcGIS and stored onboard UAV).

To verify the orthorectification results, real-world images of the UAV in turning, level, and climbing flight states were collected, as illustrated in Figure 9, Figure 10 and Figure 11. During a right turn, the UAV’s roll angle exceeded 24°, whereas the pitch angle exceeded 9° during climbing. A comparison was conducted between the corrected images and satellite reference images. The results indicate that the orthorectified UAV infrared images exhibit high consistency with the orthographic reference, thereby validating the effectiveness of the proposed method.

### 5.3. Experiments on Image Matching

**Figure 12:**
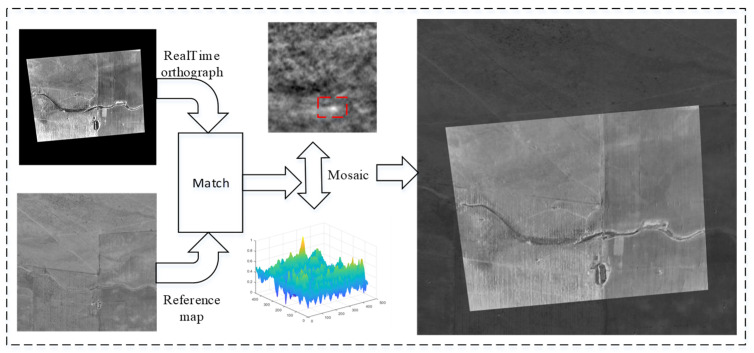
Image matching and mosaic results for UAV in roll flight.

**Figure 13:**
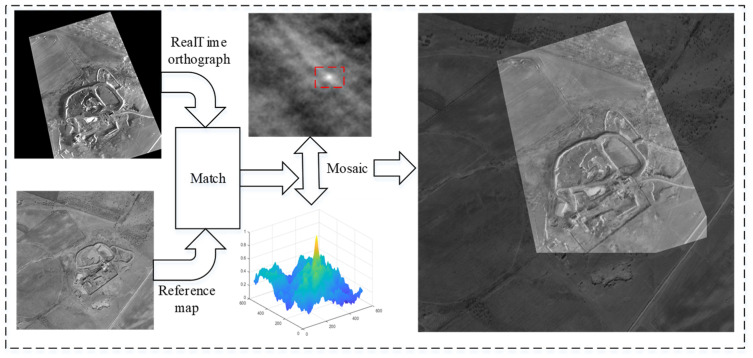
Image matching and mosaic results for UAV in level flight.

**Figure 14:**
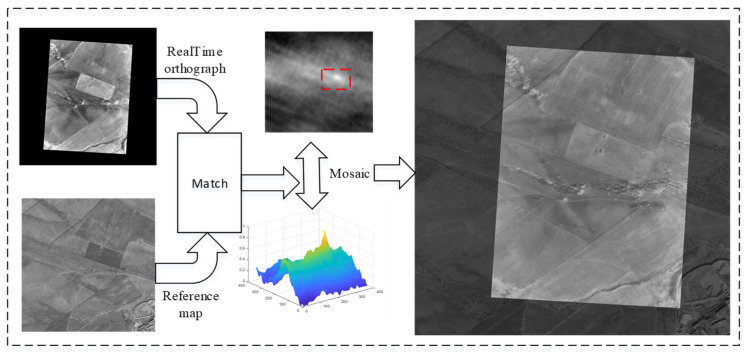
Image matching and mosaic results for UAV in climbing flight.

Figure 12, Figure 13 and Figure 14 depict the matching results of the UAV in roll, level flight, and climb states, respectively. The results demonstrate that the UAV is capable of achieving robust matching across various fixed flight conditions. Nevertheless, when the UAV experiences substantial attitude changes, image registration, while feasible, exhibits increased mosaic errors at the image edges, which consequently elevate positioning errors. Conversely, under conditions of minimal attitude variations, the accuracy of image matching and mosaicking is significantly improved.

When the image texture is weak or fails to provide extractable features, the system automatically expands the ROI search range to ensure that UAV-captured images remain within the ROI boundaries. Table 2 presents the processing time statistics for template matching, Log-Gabor filtering, orthorectification, and KF algorithms under varying ROI sizes. As shown in Table 2, with the expansion of the ROI search range, the maximum latency of the template-matching process reaches 472.39 ms, while the Log-Gabor filter exhibits a maximum latency of 207.88 ms. In contrast, the processing times of both the orthorectification and KF algorithms remain virtually unaffected by ROI size variations. Specifically, the orthorectification process requires approximately 4 ms, and the KF algorithm completes its process in less than 0.03 ms.

Since the KF algorithm operates in a separate thread from template matching, Log-Gabor filtering, and orthorectification, and the laser inertial navigation system provides real-time positioning information, image processing delays can be effectively compensated for through a time-delay compensation algorithm. Consequently, the real-time performance of the SINS/SMNS integrated navigation system depends solely on the KF processing time, which is maintained well below 1 ms, fully meeting the operational requirements of unmanned aerial vehicles.

### 5.4. Experiments on Integrated Navigation

**Figure 15:**
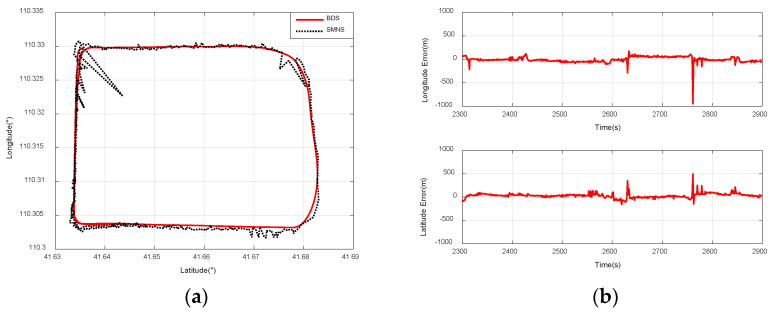
Comparison of positioning results between the SMNS and the Beidou navigation receiver during a rectangular flight path at a 3000 m relative altitude. (**a**) SMNS and Beidou positioning results. (**b**) Longitude error and latitude error.

**Figure 16:**
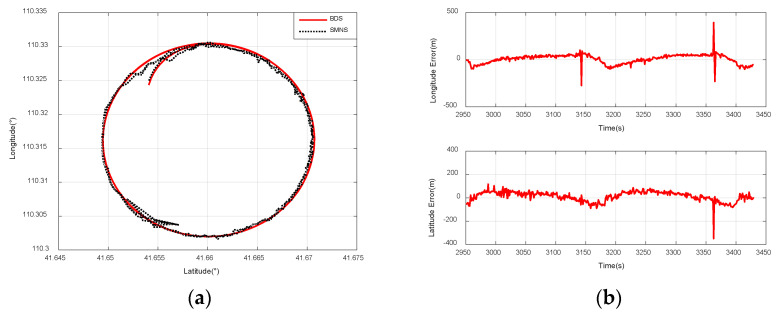
Comparison of positioning results between the SMNS and the Beidou navigation receiver during a small-radius circular flight path at a 3000 m relative altitude. (**a**) SMNS and Beidou positioning results. (**b**) Longitude error and latitude error.

**Figure 17:**
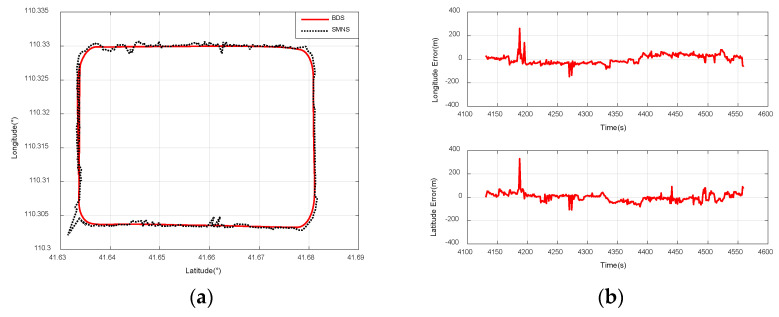
Comparison of positioning results between the SMNS and the Beidou navigation receiver during a rectangular flight path at a 1500 m relative altitude. (**a**) SMNS and Beidou positioning results. (**b**) Longitude error and latitude error.

The location of the UAV is determined using the scene-matching positioning method outlined in Section 3. Figure 15, Figure 16 and Figure 17 illustrate the scene-matching position errors for the three flight paths. The mean error $e_{mean}$ is calculated as follows:

(36) $$e_{mean}=\frac{1}{n}\sum_{i=1}^{n}\left(a_{i}-b_{i}\right),i=1,2,...,n$$

where $a_{i}$ represents the sample value and $b_{i}$ denotes the reference value.

The standard deviation $e_{std}$ of the variable $c_{i}$ is calculated by

(37) $$e_{std}=\sqrt{\frac{1}{n}\sum_{i=1}^{n}(c_{i}-e_{mean}{)}^{2}},i=1,2,...,n$$

In this study, the positional data provided by the Beidou navigation receiver serve as the reference value. Based on Equations (36) and (37), we investigate the impact of two parameters—the UAV’s flight paths and its relative flight altitude—on the positioning accuracy of the scene-matching navigation system.

Figure 15a compares the positioning results of the SMNS and the Beidou navigation receiver during a rectangular flight path at a 3000 m relative altitude. Figure 15b illustrates the positioning error curve of the SMNS during a rectangular flight path at a 3000 m ground-relative altitude.

Figure 16a compares the positioning results of the SMNS and the Beidou navigation receiver during a small-radius circular flight path at a 3000 m ground-relative altitude. Figure 16b illustrates the positioning error curve of the SMNS during a small-radius circular flight path at a 3000 m ground-relative altitude.

Figure 17a compares the positioning results of the SMNS and the Beidou navigation receiver during a rectangular flight path at a 1500 m ground-relative altitude. Figure 17b illustrates the positioning error curve of the SMNS during a rectangular flight path at a 1500 m ground-relative altitude.

The corresponding analysis results are summarized in Table 3. The following can be observed:

1. When the UAV is flying at a relative altitude of 1500 m, the positioning accuracy of the SMNS is superior to that when the UAV is flying at a relative altitude of 3000 m;
2. Regardless of the flight path of the UAV, the SMNS exhibits significant errors in positioning results. This phenomenon is particularly pronounced when the UAV undergoes changes in its maneuvering state, where the positioning errors become even more noticeable.

**Table 3:** Statistical analysis of scene-matching location errors under three operational conditions (Condition I: SMNS @3000 m; Condition II: proposed integrated navigation system @3000 m; Condition III: proposed integrated navigation system @1500 m).

Name	Conditions	Mean (m)	Standard Deviation (m)
Longitude error	Condition I	−3.63	61.96
Latitude error		31.80	48.57
Longitude error	Condition II	7.49	50.61
Latitude error		16.08	40.99
Longitude error	Condition III	−0.83	38.00
Latitude error		1.34	35.78

**Figure 18:**
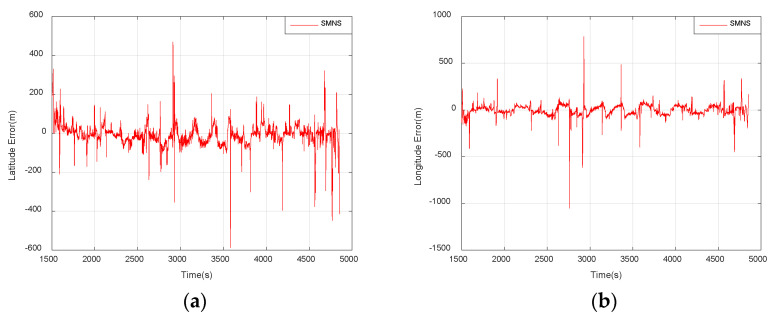
Horizontal position errors of the SMNS. (**a**) Latitude error. (**b**) Longitude error.

**Figure 19:**
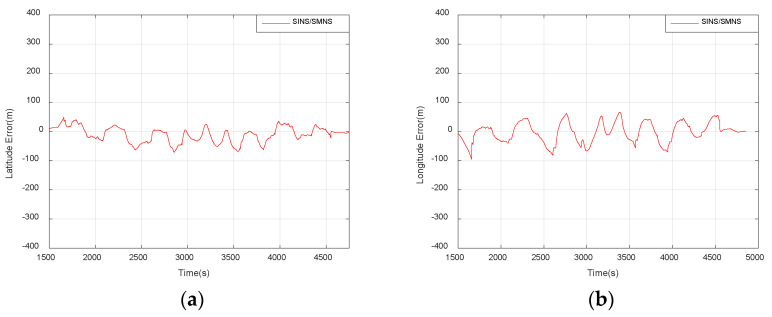
Horizontal position errors of the SINS/SMNS integrated navigation system. (**a**) Latitude error. (**b**) Longitude error.

As shown in Figure 18, the horizontal position errors of the SMNS exhibit numerous significant gross errors, which could pose a substantial risk to the safe operation of UAVs. To address this issue, we performed data fusion on the positioning results from the SMNS and the SINS. The horizontal position errors of the integrated navigation system after fusion are illustrated in Figure 19.

As shown in Table 4, the standalone SMNS exhibits latitude errors with a mean of −9.48 m and a standard deviation of 54.79 m, while showing longitude errors with a mean of −1.87 m and a standard deviation of 63.52 m.

The SINS/SMNS integrated system demonstrates improved performance:

1. Latitude error: mean = −10.41 m; std = 26.11 m.
2. Longitude error: mean = −2.33 m; std = 34.59 m.

These results indicate a 52.34% reduction in latitude error deviation and a 45.54% reduction in longitude error deviation compared to the standalone SMNS, meeting UAV operational requirements for mission execution, precision navigation, and controlled recovery in GNSS-denied environments.

## 6. Conclusions

To address the positioning challenges of unmanned aerial vehicles (UAVs) in GNSS-denied environments, we proposed an integrated navigation method based on SINS/SMNS. This approach combines image preprocessing, feature matching, and multi-sensor data fusion to achieve robust navigation. The proposed method was validated through simulations using actual flight data. The results demonstrate that the SINS/SMNS integration significantly outperforms standalone SMNS in horizontal positioning accuracy, improving latitude accuracy by 52.34% and longitude accuracy by 45.54%. The achieved accuracy meets UAV operational requirements in GNSS-denied environments.

## Figures and Tables

**Table 1:** Integrated navigation system verification platform: experimental equipment specifications and performance metrics (components: laser inertial navigation system, Beidou navigation receiver, atmospheric data sensor system, day–night electro-optical reconnaissance payload, and high-performance processor module).

Device Name	Metric Name	Parameter
Laser inertial navigation system	Gyroscope bias stability	≤0.05°/h
	Accelerometer bias stability	≤100 μg
Beidou navigation receiver	Velocity accuracy	≤0.05 m/s
	Position accuracy	≤0.1 m
Atmospheric data sensor system	Airspeed	≤1 m/s
	Barometric altitude	≤10 m
Day–night electro-optical reconnaissance payload	Visible light resolution	1920 × 1080
	Infrared resolution	1280 × 1024
	Laser ranging accuracy	5 m
High-performance processor module	GPU	Volta architecture with 512 CUDA cores
	CPU	8-core Carmel Armv8.2 64-bit CPU 32 GB
	RAM	256-bit LPDDR4
	External storage	1 TB SSD

**Table 2:** Statistical results of actual processing time for template matching, Log-Gabor filtering, orthorectification, and KF algorithms on a high-performance processor across different ROI sizes.

ROI Size (Pixel)	Template Matching (ms)	Log-Gabor Filtering (ms)	Orthorectification (ms)	KF (ms)
2000 × 2000	67.33	34.62	3.96	0.02638
3000 × 3000	126.53	54.26	4.06	0.02624
3800 × 3800	209.6123	83.51	4.07	0.02736
5400 × 5400	472.39	207.88	4.08	0.02675

**Table 4:** Statistical analysis results of the positioning errors for the integrated navigation system based on the SINS/SMNS.

Methods	Error Name	Mean (m)	Standard Deviation (m)
SMNS	Latitude error	−9.48	54.79
	Longitude error	−1.87	63.52
SINS/SMNS	Latitude error	−10.41	26.11
	Longitude error	−2.33	34.59

## Data Availability

Data are contained within the article.
